# Genome-wide ChIP-seq analysis of human TOP2B occupancy in MCF7 breast cancer epithelial cells

**DOI:** 10.1242/bio.014308

**Published:** 2015-10-12

**Authors:** Catriona M. Manville, Kayleigh Smith, Zbyslaw Sondka, Holly Rance, Simon Cockell, Ian G. Cowell, Ka Cheong Lee, Nicholas J. Morris, Kay Padget, Graham H. Jackson, Caroline A. Austin

**Affiliations:** 1 Institute for Cellular and Molecular Biosciences, Newcastle University, Newcastle upon Tyne NE2 4HH, UK; 2 The Bioinformatics Support Unit, Faculty of Medical Sciences, Newcastle University, Newcastle upon Tyne NE2 4HH, UK; 3 School of Biomedical Sciences, Newcastle University, Newcastle upon Tyne NE2 4HH, UK; 4 Department of Applied Biology, Northumbria University, Newcastle upon Tyne NE1 8ST, UK; 5 Institute for Cellular Medicine, Newcastle University, Newcastle upon Tyne NE2 4HH, UK

**Keywords:** ChIP seq, TOP2B, Topoisomerase II

## Abstract

We report the whole genome ChIP seq for human TOP2B from MCF7 cells. Using three different peak calling methods, regions of binding were identified in the presence or absence of the nuclear hormone estradiol, as TOP2B has been reported to play a role in ligand-induced transcription. TOP2B peaks were found across the whole genome, 50% of the peaks fell either within a gene or within 5 kb of a transcription start site. TOP2B peaks coincident with gene promoters were less frequently associated with epigenetic features marking active promoters in estradiol treated than in untreated cells. Significantly enriched transcription factor motifs within the DNA sequences underlying the peaks were identified. These included SP1, KLF4, TFAP2A, MYF, REST, CTCF, ESR1 and ESR2. Gene ontology analysis of genes associated with TOP2B peaks found neuronal development terms including axonogenesis and axon guidance were significantly enriched. In the absence of functional TOP2B there are errors in axon guidance in the zebrafish eye. Specific heparin sulphate structures are involved in retinal axon targeting. The glycosaminoglycan biosynthesis–heparin sulphate/heparin pathway is significantly enriched in the TOP2B gene ontology analysis, suggesting changes in this pathway in the absence of TOP2B may cause the axon guidance faults.

## Abstract (precis)

**Summary:** Gene ontology enrichment analysis of genes associated with human TOP2B peaks, identified by whole genome ChIP seq used to identify regions of binding, highlighted a number of processes in neuronal development including axonogenesis and axon guidance.

## INTRODUCTION

The topoisomerase family of nuclear enzymes catalyse the interconversion of different topological forms of DNA. Two type II topoisomerase isoforms are present in vertebrate cells, TOP2A (topoisomerase IIα) and TOP2B (topoisomerase IIβ) (Austin and Marsh, 1998). The TOP2 catalytic mechanism involves the simultaneous cleavage of both DNA strands of a double-stranded DNA segment, transiently leaving the 5′-ends covalently attached to the dimeric topoisomerase enzyme via 5′-phosophotyrosine linkages to each monomer. A second DNA duplex can then pass through the enzyme-bridged DNA gate in a strand passage reaction, prior to religation of the break. From previous studies using *in vitro* DNA cleavage and footprinting analysis it was shown that TOP2 protects a region of 25 (*Drosophila* TOP2) or 28 (mammalian TOP2) nucleotides, with the TOP2 DNA cleavage site in the centre of the protected region (Lee et al., 1989; Thomsen et al., 1990). Crystal structures of the cleavage and re-joining domains of TOP2A and TOP2B complexed to nicked oligonucleotide substrates reveal TOP2:DNA complexes containing symmetrical dimers of TOP2, confirming previous biophysical studies (Wendorff et al., 2012; Wu et al., 2011). TOP2B displays an apparent dissociation constant for linear DNA of 130 nM assessed by electrophoretic mobility shift analysis (EMSA) (West and Austin, 1999). By comparison, the transcription factor SP1 displayed an EMSA-derived K_D_ of 0.4 nM for a consensus DNA binding site (Letovsky and Dynan, 1989). Beyond these *in vitro* studies, the recruitment and binding of human TOP2 to specific loci in the context of chromatin is poorly understood.

TOP2A is required for chromatin condensation and chromosome segregation and is essential in eukaryotic cells (Akimitsu et al., 2003). By contrast, TOP2B is not essential for human or mouse cells in culture (Dereuddre et al., 1997), but *Top2b^−/−^* mice are non-viable. *Top2b* null embryos exhibit no gross abnormalities in the morphology of major organs during development, but exhibit a defect in neural development where motor axons fail to form contacts with skeletal muscles and sensory axons do not enter the spinal cord, although neurogenesis appears to be normal. Failure to innervate the diaphragm, resulting in an inability to breath is thought to be the cause of perinatal lethality (Yang et al., 2000). Furthermore, cultured neurons lacking Top2b produced shorter neurites than wild type neurons, suggesting Top2b is involved in the process of neurite outgrowth (Nur-E-Kamal et al., 2007). The nature of the defects observed in *Top2b^−/−^* cells suggests a possible role for Top2b in gene expression programs required for late neural development, and expression analysis in murine brain revealed that about a third of developmentally regulated genes exhibit altered expression (mostly exhibiting reduced expression) in *Top2b^−/−^* mice compared to wild-type siblings. Top2b was shown to be present in the 5′ and upstream regions of a number of Top2b-sensitive genes, suggesting a direct role of Top2b in transcription of these genes, but not genes that were insensitive to Top2b (Lyu et al., 2006; Tiwari et al., 2012). The requirement for Top2b during neural development is consistent with a previous finding that Top2b mRNA levels significantly increase in murine neonatal brain (Capranico et al., 1992). It appears that Top2b becomes crucial as neural cells enter the postmitotic stage, when Top2a is down regulated (Tiwari et al., 2012). Interestingly, Top2b represses the neurotrophin gene (*Ngfr p75*) and up-regulation of this gene has been implicated in cell death of *Top2b^−/−^* neurons. A cre-lox *Top2b^−/−^* murine model demonstrated that neurons lacking Top2b had defective positioning in the cerebral cortex (Lyu and Wang, 2003). A role for Top2b in neural development is further supported by studies in rat. RNA *in situ* analyses in rat brain at P10 (Tsutsui et al., 1993) shows high level expression in the cerebellum and *Top2b* has been shown to be involved in granule cell differentiation (Tsutsui et al., 2001). Top2b DNA complexes were isolated from rat cerebellar granule cells by chromatin immunoprecipitation (ChIP) followed by microarray analysis (ChIP on chip) demonstrating that Top2b is associated with genes essential for neuronal maturation and that Top2b-dependent genes encoded long transcripts (Lyu et al., 2006; King et al., 2013; Sano et al., 2008).

In zebrafish, top2a is maternally encoded and is essential for development (Amsterdam et al., 2004; Dovey et al., 2009; Sapetto-Rebow et al., 2011). In contrast, top2b is not maternally encoded and expression commences after the mid blastula transition becoming widely expressed in the developing embryo. Consistent with a conserved role for top2b in neural development, the blind *notorious* (*noto*) mutant isolated in an ENU forward genetic screen is characterised by neurite targeting defects in retinal ganglion cell axons and dendrites and is caused by a mutation in top2b (Nevin et al., 2011).

TOP2B expression during human development has been analysed by *in situ* analysis (Zandvliet et al., 1996). Human TOP2B protein directly interacts with a number of proteins including CD3ε UBC9, TOPBP1, p53, pRB, SNF2H, HDAC1 and HDAC2 (Cowell et al., 2000; Johnson et al., 2001; LeRoy et al., 2000; Mao et al., 2000; Nakano et al., 1996; Tsai et al., 2000; Xiao and Goodrich, 2005; Yamane et al., 1997; Yuwen et al., 1997) several of which are involved in transcriptional regulation. Inhibition of HDACs by TSA redistributes TOP2B from heterochromatin to euchromatin in mouse epithelial cells (Cowell et al., 2011). TOP2B is also found associated with several complexes involved in the regulation of transcriptional initiation. It is a component of the Groucho/TLE1 co-repressor complex, alongside a PARP sensor which triggers a switch from neurogenic gene repression to neurogenic activation mediated by CaMKinase IIδ (Ju et al., 2004). TLE1 also promotes neuronal survival of post mitotic neurons, in combination with FoxG1 (Dastidar et al., 2012), consistent with the suggestion that murine Top2b is involved in preventing the premature death of post mitotic neurons (Tiwari et al., 2012).

TOP2B is involved in ligand mediated transcription in response to estradiol, androgen, retinoic acid, thyroid hormone or TPA (Haffner et al., 2010; Ju et al., 2006), and in response to insulin in liver cells in the induction of fatty acid synthase (*FAS*) expression (Wong et al., 2009). In each of these examples, it has been demonstrated that the inducing agent (steroid hormone ligand or insulin) leads to the recruitment of TOP2B along with DNA-PK and PARP to the 5′ region of the gene in question and the formation of a promoter DNA double-strand break that is presumed to be induced by TOP2B. In the case of the *FAS* gene it has been suggested that the DSB serves to activate DNA-PK to then modulate one or more further promoter-bound factor(s) via phosphorylation. It is likely that in at least the case of *FAS*, TOP2B interacts with DNA-PK since in the absence of DNA-PK TOP2B is not recruited to chromatin (Wong et al., 2009). Ligand-dependent chromatin recruitment of TOP2B with 17β-estradiol was confirmed in MCF7 cells (Ju et al., 2006; Cowell et al., 2012) and RA induced recruitment of TOPB to the 5′-region of the *RARB*, was reported by McNamara et al. (2008), who showed that TOP2B-mediated repression of RA-induced genes could lead to RA resistance in APL cell lines. Thus, TOP2B is recruited to various promoter regions in response to nuclear hormones and insulin and is required for the completion of neural development. TOP2B is also reported to be involved in transcriptional elongation (King et al., 2013) especially of long genes. Topoisomerases also have a role in determining chromatin supercoiling over larger distances, in the form of supercoiling domains which are defined as alternating domains of relative over- or under-winding of DNA (Naughton et al., 2013).

To further study the recruitment of TOP2B to chromatin in response to a signal such as steroid hormone ligand binding, we set out to determine TOP2B genomic distribution and its changes upon estradiol stimulation using ChIP-seq in MCF7 cells. Since the studies described above (Ju et al., 2006; Cowell et al., 2012; Perillo et al., 2008) had demonstrated localised recruitment of TOP2B to specific chromatin regions of genes analysed by ChIP analysis, we hypothesised that TOP2B chromatin distribution would appear as peaks of increased binding at the resolution achieved by ChIP-seq. Therefore, we employed three peak-calling methods (including manual inspection) to identify regions of localised TOP2B occupancy. In control and estradiol-treated cells 2872 and 3265 peaks were identified by two or more of the peak finding methods employed. Surprisingly, TOP2B peaks were less frequently coincident with transcription-associated features after estradiol treatment, suggesting a role of TOP2B in repression of transcription. Gene ontology enrichment analysis of genes associated with TOP2B peaks highlighted a number of processes in neuronal development including axonogenesis and axon guidance.

## RESULTS

### Genome wide identification of TOP2B sites of occupancy in MCF7

**Fig. 1.**
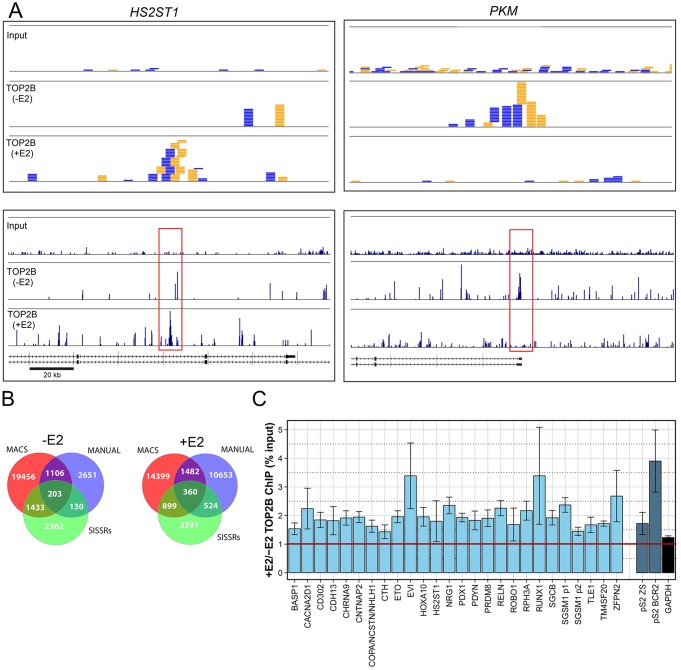
**TOP2B ChIP-seq peak identification.** Peaks of TOP2B occupancy were identified by manual inspection of aligned sequence reads and by MACS and SISSRS. (A) Example TOP2B peaks identified by manual analysis. Top forward (blue) and reverse (amber) 36 base sequence reads corresponding to peaks identified in the *HS2ST1* and *PKM* genes. Tracks are shown for Input and TOP2B ChIP derived from untreated (−E2) and estradiol-treated (+E2) cells. Bottom, larger genomic region centred on the peak showing average base density/coverage of reads, a 20 kb scale bar is shown. (B) Venn diagram representing intersects in sets of peaks called by the three analysis methods for untreated and E2-treated cells respectively. (C) Quantitative TOP2B ChIP PCR validation of 26 examples of peak regions identified by two or more peak calling methods the ratios of the +E2/−E2% input values are shown (light blue bars). Data for the E2-responsive pS2 promoter are also shown (dark blue bars). All ChIP experiments included an AcH3 GAPDH ChIP, the average +E/−E ratio for this is also shown (black bar). Data is presented as mean±s.e.m.

ChIP-seq was carried out to determine the sites of occupancy of TOP2B in human MCF7 cells. Since previous work had suggested a role for TOP2B in the transcriptional regulation of specific genes in response to estradiol (Ju et al., 2006; Perillo et al., 2008), we carried out chromatin immunoprecipitation (ChIP) from cells treated with 17β-estradiol (E2, 10 nM, 30 min), and untreated cells. Recovered DNA was subject to single-end sequencing on an Illumina Genome Analyser II. Sixty seven million TOP2B ChIP-Seq reads were aligned to the human reference genome. As TOP2B has a lower affinity for DNA than sequence specific transcription factors for which ChIP was previously used, TOP2B lacks their high DNA sequence specificity it was unclear how best to analyse the sites of binding of TOP2B. Therefore we initially carried out a visual manual inspection of aligned sequence reads using Genome Studio software (2008.1 Framework). Employing input as controls revealed the presence of peaks in the distribution of aligned TOP2B reads. Two example peaks are shown in Fig. 1A one with estradiol on *HS2ST1* and one without estradiol on *PKM*. Subsequently, two computational peak finding methods were utilised, the Model-based Analysis of ChIP-Seq (MACS) peak calling algorithm (Zhang et al., 2008) and Site Identification from Short Sequence Reads (SISSRs) (Jothi et al., 2008). For MACs and SISSRs sequence tags derived from input chromatin were used to control for amplified regions in MCF-7 cells. Thus, three peak sets were produced (manual, MACs and SISSRs) for E2-treated MCF7 cells and three peak sets for untreated cells. These TOP2B peaks indicate occupancy sites for TOP2B in the genome and merging all three peak sets gave 27,341 and 30,908 peaks, (merged peak sets) from untreated and E2-treated MCF7 cells respectively. The most stringent peaks were defined as those identified by all three methods. These numbered 203 from untreated cells and 360 from E2-treated cells. This subset included approximately 1% of the peaks, whilst over 9% of the peaks were identified by two methods. Thus more than 10% of the peaks were found by at least two peak calling methods; 2872 and 3265 for untreated and E2-treated cells respectively (Fig. 1B). The high confidence sets of TOP2B peaks called by at least two methods were used for further analysis unless otherwise indicated and we refer to these sets as TOP2B^2^ peak sets from hereon in. Twenty six peaks were validated by ChIP-qPCR and the data obtained for these in untreated and E2-treated cells was calculated as a percentage of input, the ratio of the signals for untreated and estradiol treated cells is shown in Fig. 1C.

### Genomic location of TOP2B peaks

**Fig. 2.**
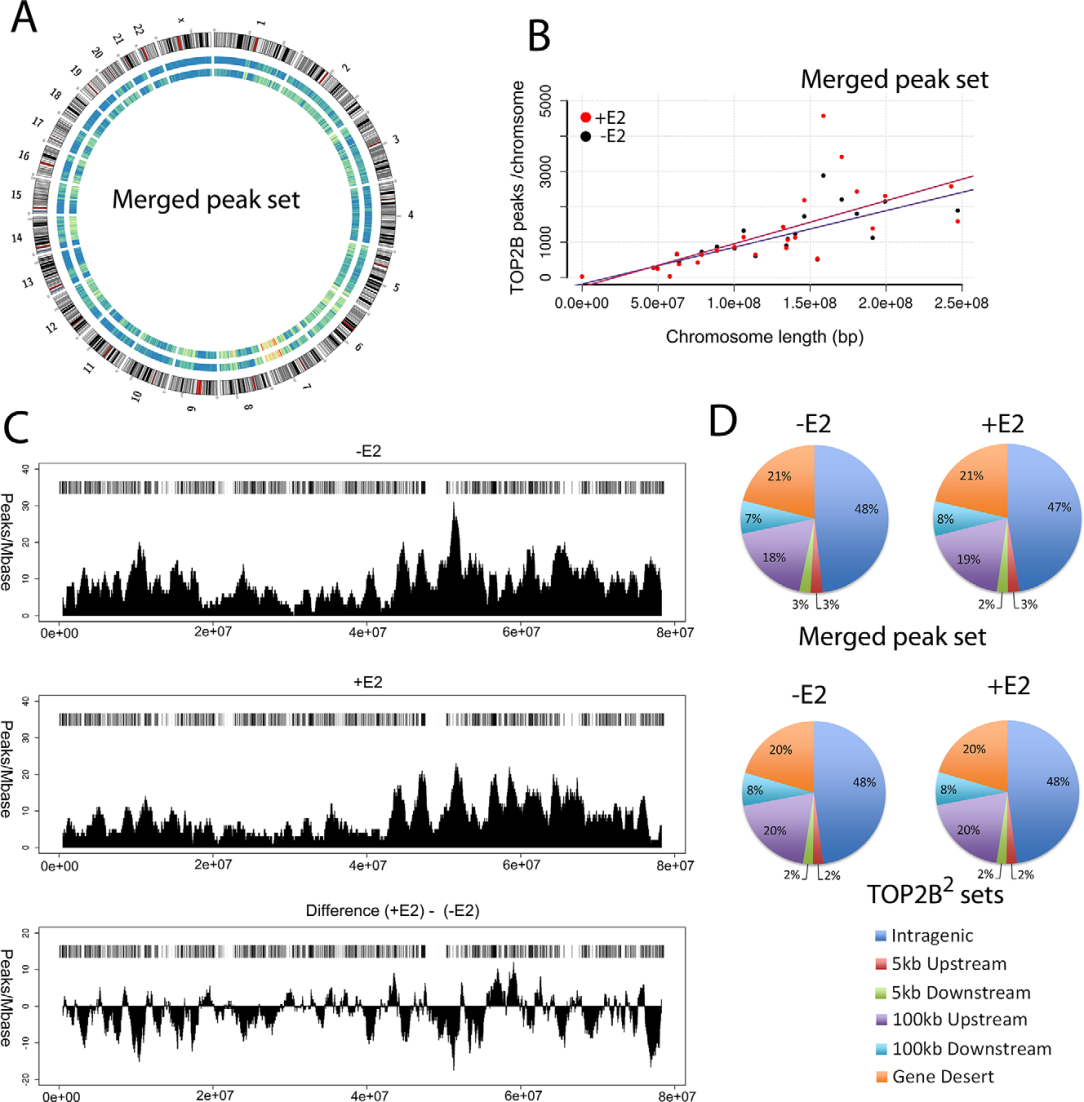
**Global distribution of TOP2 peaks.** (A) Circle plot displaying TOP2B peak density for all chromosomes. Outer circle, chromosome G-banding ideogram; middle circle, TOP2B peak density for untreated cells; inner circle, TOP2B peak density for E2-treated cells. The colours in A come from an ‘R color brewer’, an eleven division reversed spectral palette was used, from blue to red. Blue is the lowest and red the highest. Peak density plots and the data in (B) are derived from the ‘merged peak sets’ containing all of the peaks identified by each of the peak calling methods. (B) Correlation of peak number with chromosome length. (C) Distribution of TOP2B peak density across a representative chromosome (Chr17) for E2-treated and untreated cells with gene density (horizontal bar). (D) Global distribution of TOP2B peaks in relation to genes for the ‘merged peak sets’ (see above) and the TOP2B^2^ sets, corresponding to peaks called by any of the peak calling methods or by two methods, respectively.

The distribution of the merged sets of TOP2B peaks identified by manual, MACs and SISSRs (27,341 and 30,908) across the genome from untreated and E2-treated cells are shown in a circle diagram in Fig. 2A. TOP2B peaks were observed across the whole genome, with a slightly higher density on chromosomes 6 and 7 (Fig. 2A). Apart from chromosomes 6 and 7 the number of peaks on a chromosome is proportional to chromosome length under both treatments (Fig. 2B). Interestingly some chromatin regions become more- or less peak-rich upon the treatment suggesting that TOP2B occupancy on the chromatin is dynamic, to illustrate this the peak distribution with and without estradiol on chromosome 17 is shown (Fig. 2C). The location of the TOP2B peaks relative to genes was determined globally for the combined set of peaks obtained by any calling method (merged peak set), and for the TOP2B^2^ peak sets (those peaks called by at least two methods) (Fig. 2D). TOP2B peaks were assigned one of the following categories – peak on a gene, (intragenic, TSS to the end of the mRNA transcript); peak in a promoter region, (5 kb upstream of a TSS); peak at the 3′ end of a gene, (5 kb downstream of the gene); or within 100 kb of the 5′ end of a gene (100 kb upstream) or within 100 kb of the 3′ end of a gene (100 kb downstream) or more than 100 kb from a gene (gene desert). Analysis of both peak sets yielded very similar percentages of peaks in each category in the presence or absence of ligand, (Fig. 2D). Approximately 75% of both data sets are within 100 kb of a gene. 100 kb was used as ERα has been shown to influence gene expression up to 100 kb (Lin et al., 2007; Carroll et al., 2006). In both treatments 48% of the TOP2B peaks were found on genes and 2% within 5 kb upstream. Thus, half of the TOP2B peaks are found on a gene or within 5 kb of a TSS. This is comparable to the percentage of ERα binding sites found on a gene or within 5 kb of the TSS, average 48% (Lin et al., 2007; Joseph et al., 2010).

**Fig. 3.**
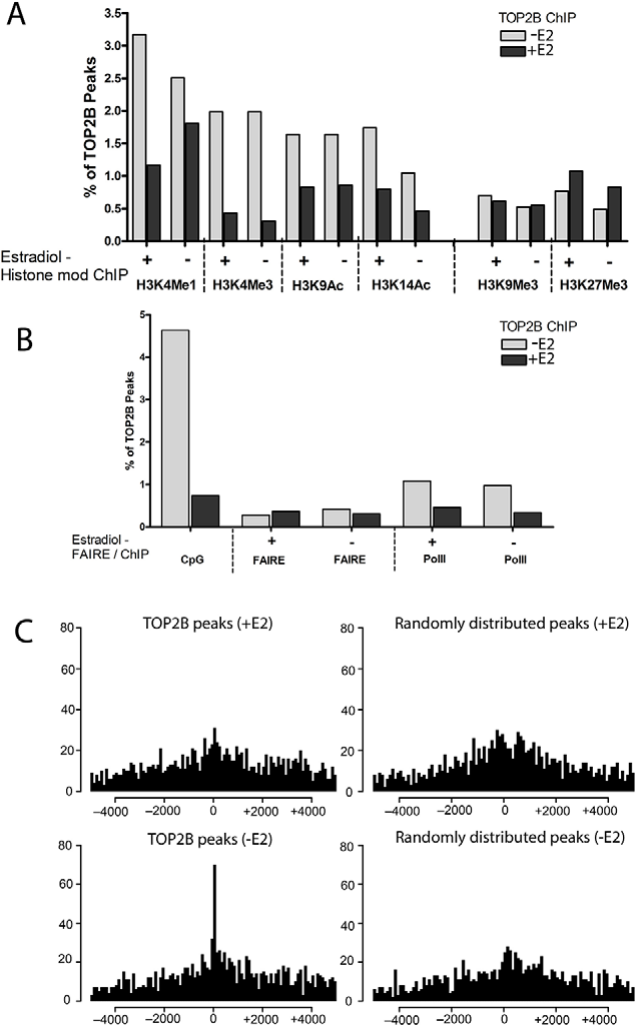
**Estradiol treatment reduces the frequency of TOP2B occupancy at sites associated with transcriptional activation markers.** (A) A smaller proportion of TOP2 peaks coincide with epigenetic markers of transcriptional activation in E2-treated than in untreated MC7 cells. (B) Estradiol treatment leads to a pronounced reduction in TOP2B peaks that coincide with CpG islands and sites of RNA pol II occupancy but not with accessible chromatin regions (FAIRE). (C) Effect of estradiol treatment on global distribution of distances of TOP2B peaks from transcription start sites. Data represented in A-C were derived from the TOP2B^2^ sets. Genomic locations enriched for the histone modifications and other features shown in control or estradiol treated (10 nM, 3 h) cells were obtained from Gene Expression Omnibus, as detailed in the legend to Fig. S1. In A and B histone modification and RNA pol II Chip and FAIRE data correspond to untreated and estradiol treated cells as indicated below the histograms. TOP2B ChIP without estradiol is shown in grey, TOP2B ChIP with estradiol is shown in black. The data it was compared to was determined in the presence or absence of estradiol as indicated by + or −.

Next we identified TOP2B peaks that coincided with transcriptional markers. The location of TOP2B^2^ peaks were analysed for coincidence with histone markers of transcriptional activation (H3K4me1, H3K4me3, H3K9ac, and H3K14ac) or transcriptional repression (H3K9me3 and H3K27me3) (Joseph et al., 2010), with RNA pol II ChIP, open chromatin (FAIRE) and with CpG islands (Fig. 3). Estradiol treatment decreased the proportion of TOP2B peaks associated with transcriptional activation markers; the largest decrease (4.7 fold) was observed at the H3K4me3 sites which are associated with active promoters. In the absence of estradiol ∼2% of the TOP2B^2^ peaks were coincident with H3K4me3 marks and this reduced with estradiol treatment. In contrast following estradiol treatment the proportion of TOP2B peaks at H3K27me3 sites associated with transcriptional repression increased slightly (Fig. 3A). Coincidence between CpG islands and the TOP2B peaks was determined by comparison to the CpG island track in UCSC. In the untreated cell data set 4.63% of the TOP2B peaks coincided with a CpG island (133 peaks). Following exposure to estradiol for 30 min only 0.74% of the TOP2B peaks coincided with a CpG island (24 peaks) (Fig. 3B). There are estimated to be 45,000 CpG islands in the human genome (Antequera and Bird, 1993), roughly 1.5% of the genome is made up of CpG islands, therefore CpG islands are over represented (4.63%) in the TOP2B peak data set from untreated MCF7 cells and underrepresented (0.74%) in the TOP2B peak data set from MCF7 cells treated with estradiol. E2 treatment also resulted in reduced association of TOP2B peaks with RNA pol II. However, unlike the reduced association with transcription-associated histone modification features, E2-treatment did not change the proportion of TOP2B peaks associated with open chromatin, as determined by FAIRE (Fig. 3B; Fig. S2).

In the converse analysis to determine which histone modifications co-localise with TOP2B peaks, estradiol substantially reduced the percentage of each of the transcription-associated epigenetic features (except FAIRE) and CpG islands associated with TOP2B peaks (Fig. S2). For example ∼0.2% of the H3K4Me3 peaks co localised with TOP2B^2^ peaks in the absence of estradiol and this reduced 4.1 fold with estradiol. Fig. 3C shows the distribution of TOP2B^2^ peaks relative to the nearest TSS. Upon treatment with estradiol there is a reduction in the number of TOP2B peaks near transcription start sites. In the TOP2B^2^ set 4.84% (139/2872) of peaks in the untreated data set are within 1 kb either side of a TSS compared to 2.97% (97/3265) of the peaks in the E2-treated data set (Fig. 3C). Thus, the distribution of TOP2B peaks changes in response to estradiol, with a decreased proportion of TOP2B peaks coincident with H3K4me3 (Fig. 3A), CpG islands and RNA pol II sites (Fig 3B) and fewer TOP2B peaks near TSSs in the presence of E2 (Fig. 3C).

### Gene ontology for genes associated with TOP2B peaks

The TOP2B^2^ peaks were intersected with the UCSC known genes database to identify the genes bearing TOP2B peaks (Table S2). Gene ontology enrichment analysis was carried out using GOstats Bioconductor package, using a hypergeometric test (Falcon and Gentleman, 2007) to identify cellular components, biological processes and molecular functions that are carried out by the proteins encoded by the genes. These are displayed as heatmaps in Fig. S3, where an FDR cut off of <0.01 was used.

The most enriched cellular components included neuron part, neuron projection, cell projection and dendrite, consistent with the known association of TOP2B with neuronal development reported in mouse, rat and zebrafish development (Yang et al., 2000; Nur-E-Kamal et al., 2007; Tiwari et al., 2012; Capranico et al., 1992; Lyu and Wang, 2003; King et al., 2013; Sano et al., 2008; Nevin et al., 2011).

Biological processes involved in neuronal development were also significantly over-represented in both the untreated and E2 treated data sets. Terms over-represented in the untreated data set but not in the treated set included protein auto-phosphorylation and cellular response to stimulus, those overrepresented only in the E2 treated dataset included glutamate receptor signalling and negative regulation of signal transduction.

The enriched molecular functions included signalling processes. Protein tyrosine kinase activity in the absence of estradiol but not its presence. Protein binding, calmodulin binding, ion channel activity, GTPase regulator activity and glutamate receptor activity were most significantly enriched in the E2-treated set, consistent with the previously reported functions of estradiol (Hedges et al., 2012; Meitzen and Mermelstein, 2011).

### KEGG pathways significantly enriched for genes associated with TOP2B peaks

When the strongest TOP2B peaks, those called by all three peak finding methods were analysed, the KEGG pathway glycosaminoglycan biosynthesis–heparan sulphate/heparin pathway was significantly enriched (FDR 0.001169266). Heparan sulphate is a long unbranched polysaccharide which is covalently attached to various proteins (heparin sulphate proteoglycans) on the cell surface and in the extracellular matrix. A TOP2B peak was observed by all three peak calling methods on the gene encoding heparan sulfate 2-O-sulfotransferase 1 (HS2ST1), shown in Fig. 1A. HS2ST is an enzyme 2-O-suphurotransferase that transfers sulphate to the 2 position of the iduronic acid residue of heparan. Specific combinations of 2-O- and 6-O- sulphate groups on heparin sulphate are required for retino-tectal axon targeting (Irie et al., 2002). In TOP2B^2^ set, further genes involved in this pathway appear in the peak list including *HS3ST5*, *HS6ST2*, *HS6ST3*, *NDST3* and *EXT2*.

A number of KEGG pathways were significantly enriched with the TOP2B^2^ sets. Axon guidance is significantly enriched in both E2-treated and untreated cells (FDRs 1.110957e-03 and 7.741285e-05). At least 20 genes in the KEGG pathway for axon guidance have TOP2B peaks associated with them, these include the netrins, *NTNG1* and *NTNG2*, and the netrin receptor *UNC-5*; ephrin receptors, *EPHA* and *EPHB*, and the membrane bound tyrosine kinase *FYN*; *SLIT3* and its receptor *ROBO1* (roundabout axon guidance receptor homolog 1); *SEMA3A*, *SEMA3B* and their co-receptor proteins *PLXNs* and *MET*. The intracellular effectors include GTPases and their associated proteins and kinases. These include three Rho GTPase regulating proteins ARHGAP13 (SRGAP), ARHGAP26 (FAK), and ARHGEF27 (NGEF), also RHOD, a Rho GTPase and ROCK, a Rho associated protein that acts as a downstream effector of Rho. The kinases included two serine-threonine kinases, PAK that links RHO GTPases to the cytoskeleton and nuclear signalling; and GSK3B, a glycogen synthase kinase subfamily member. Tyrosine kinases included FYN, a membrane bound non-receptor protein tyrosine kinase and FRK, a nuclear tyrosine kinase that negatively regulates proliferation. Other genes involved in axon guidance with TOP2B peaks are *AGAP3, GNA1, NCK2, CALN* and *ABLIM1*. Enrichment for the KEGG axon guidance pathway is consistent with TOP2B's previously reported role in axon guidance (Yang et al., 2000; Nur-E-Kamal et al., 2007; Lyu and Wang, 2003; Nevin et al., 2011; Heng et al., 2012).

Three other KEGG pathways are significant in the TOP2B^2^ set from untreated cells, focal adhesion (1.300159e-04), glioma (9.174241e-03), and the ErbB signaling pathway (9.770330e-03). A number of signalling proteins are enriched in these three pathways, PKC, PI3K and SHC. PAK is present in focal adhesion and ERBB signalling as well as in axon guidance.

In the presence of estradiol, in addition to axon guidance, calcium signalling and three cardiomyopathy pathways are significantly enriched. Calcium signalling pathway (5.857564e-04), arrhythmogenic right ventricular cardiomyopathy (ARVC) (6.770581e-06), dilated cardiomyopathy (4.601454e-04) and hypertrophic cardiomyopathy (HCM) (2.018838e-03). The three cardiomyopathies share a number of components associated with TOP2B^2^ peaks; these include dystrophin (DMD), integrins, and the sarcoglycan complex. Several calcium ion channels involved in cardiomyopathies are associated with TOP2B peaks including the voltage-dependent calcium channel CACNA1C, the sarcoplasmic reticulum calcium release channel RYR1, and the Na^+^/Ca^+^ exchanger and calcium anti-porter SLC8A1. The latter is also present in the calcium signalling pathway.

### Overlap between genes associated with TOP2B peaks and genes implicated in autism and schizophrenia

**Fig. 4.**
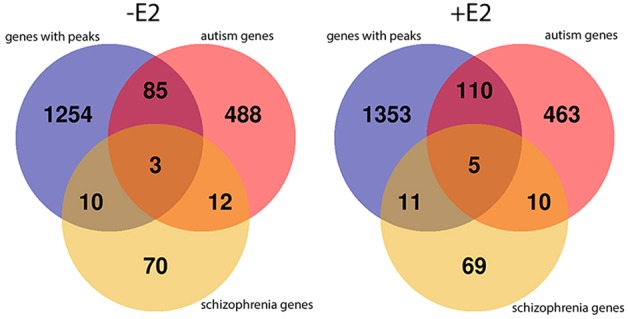
**A subset of TOP2B peaks coincide with autism and schizophrenia-associated genes.** The gene lists for this Venn diagram are shown in Table S2.

The role of TOP2B in neural development suggests it may be involved in neurodevelopmental disorders such as autism and schizophrenia. Genes associated with TOP2B peaks and genes on or near loci implicated in autism or schizophrenia (Basu et al., 2009; De Rubeis et al., 2014; Schizophrenia Working Group of the Psychiatric Genomics Consortium, 2014), have been compared, see Venn diagram in Fig. 4, the genes are listed in Table S2. TOP2B^2^ peaks from untreated cells are associated with three genes (*CNTN4*, *IMMP2L* and *SATB2*) implicated in both autism and schizophrenia whilst TOP2B^2^ peaks from E2-treated cells are associated with five genes (*CNTN4*, *IMMP2L*, *EPC*, *SMG6* and *CACNA1C*). *CACNA1C* is a calcium channel with strong links to autism and schizophrenia (Bhat et al., 2012; Giusti-Rodríguez and Sullivan, 2013), it is also implicated in cardiomyopathies. *CNTN4* and *IMMP2L* come up in both peak sets. Contactin CNTN4 is a glycosylphosphatidylinositol-anchored membrane protein that functions in neuronal plasticity and IMMP2L is an inner mitochondrial membrane peptidase-like mitochondrial protein involved in processing the signal peptide sequences to direct mitochondrial proteins to the mitochondria. IMMP2L has also been implicated in Tourette's syndrome (Bertelsen et al., 2014; Gimelli et al., 2014).

### Sequence motifs within the TOP2B peaks

**Fig. 5.**
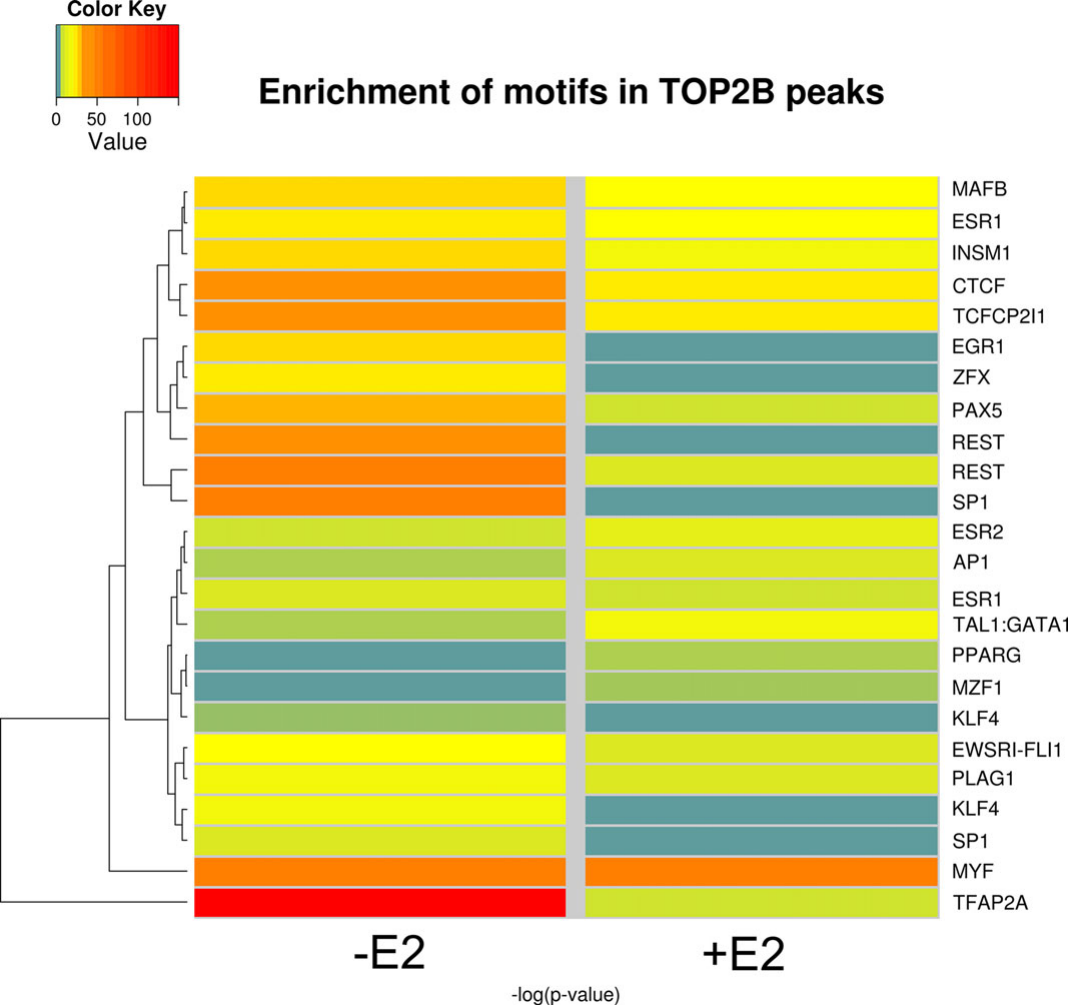
**Relative enrichment of transcription factor binding motifs associated with TOP2B peaks in untreated and E2-treated cells.** A negative logarithm of the FDR corrected *P*-value for the motif enrichment. Motifs that are not significantly enriched are blue, colour change from yellow to red depicts the increase in significance of enrichment.

To determine whether TOP2B co localises with site specific transcription factors, sequence motifs from the JASPAR database of transcription factor binding profiles (Mathelier et al., 2013) were identified in the sequences within the TOP2B^2^ peak set. ESR1 and ESR2 motifs were present and significantly enriched compared to random. Twenty three mammalian transcription factor binding motifs were enriched in the TOP2B^2^ untreated peak set and these were associated with eighteen transcription factors. Their levels of enrichment are shown as a heat map in Fig. 5 and the motifs in Fig. S4. Eighteen motifs were enriched in TOP2B^2^ E2-treated peak set and these motifs were associated with fifteen transcription factors. Motifs for the following five transcription factors were only significantly enriched in the absence of estradiol, SP1, KLF4, ERG1, PLAG1, and ZFX, whilst motifs for MZF1 and TAL1-GATA were only significantly enriched in the presence of estradiol. The remaining transcription factor binding motifs were enriched in the presence or absence of estradiol. These factors were TFAP2A, MYF, REST, CTCF, TCFCP2l1, PAX5, INSM1, MAFB, ESR1, ESR2, EWSRI-FLI1, AP1 and PPARG. It is possible that factors that only reached significance in the absence of estradiol SP1, KLF4, EGR1, PLAG1 and ZFX, or in the presence of estradiol MZF1 and TAL1:GATA1 may be responsible for the differential transcription in the presence and absence of estradiol.

**Fig. 6.**
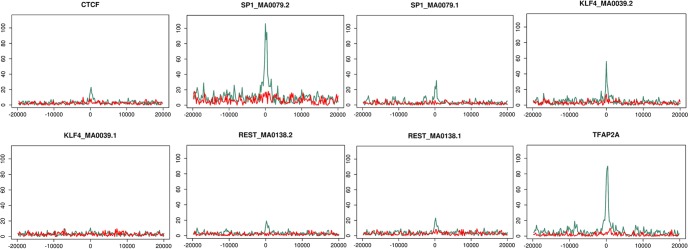
**Distribution in relation to TSSs of transcription factor motifs found within TOP2B peaks in untreated (green) and E2-treated cells (red).** The number of peaks co-localized with enriched transcription factor motifs depending on their distance from a TSS in untreated cells (green) or E2-treated cells (red).

Peaks with enriched transcription factor sequence motifs were plotted against distance from a transcription start site, up to 20 kb either side of a TSS. Fig. 6 shows plots for peaks bearing TFAP2A, SP1, KLF4, REST and CTCF motifs, further peaks with enriched transcription factor motifs are shown in Fig. S5. In most cases they display a trend to occur in the proximity of a TSS in the untreated cell set (shown in green), with a higher preference for a downstream rather than an upstream localisation (Fig. 6; Fig. S5). Peaks within 500 bases of a transcription start site were analysed further, 108 peaks were within 500 bases of a TSS in the untreated set and 57 in the E2-treated set. Of these peaks 100 in the untreated set and 45 in the E2-treated set spanned at least one of the enriched transcription factor motifs. These genes are plotted versus enriched transcription factors in heat maps and are shown in Fig. S6. The shaded blocks in the heat map show that some genes have binding sites for several of the enriched transcription factors. Transcription occurs in transcription factories and it has been suggested that these factories may come in different flavours depending upon which transcription factors are present (Papantonis and Cook, 2013).

**Fig. 7.**
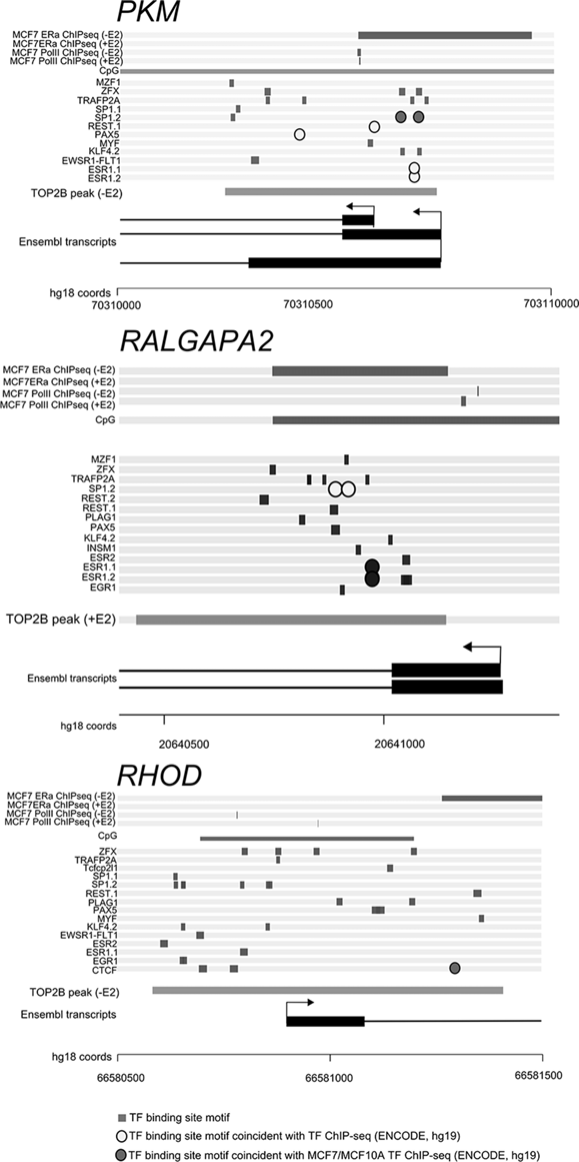
**Configuration of several TOP2B peaks that coincide with gene promoters.** For each gene: from the top, ChIP-seq for ERα and RNA Pol II (peaks shown as horizontal bars), position of any CpG island, position of transcription factor motifs (from the list of overrepresented motifs listed in Fig. S4), position of the TOP2B peak (horizontal bar), gene diagram (UCSC transcripts) and hg18 genomic coordinates.

Three gene promoters on *PKM*, *RALGAPA2* and *RHOD* have TOP2B peaks with enriched transcription factor motifs are shown schematically in Fig. 7. These three TOP2B peaks also overlap ChIP seq signals for ERα and RNA pol II and are on CpG islands. The peak on the promoter of *PKM* (pyruvate kinase, muscle) in the absence of estradiol is shown in Fig. 1. The TOP2B peak on the *PKM* promoter is coincident with a ChIP seq peak for ERα binding and an RNA pol II ChIP seq peak in MCF7 cells in the absence of estradiol, all three of these ChIP binding areas are within a CpG island. Twelve of the 23 enriched transcription factor motifs are found within the TOP2B peak sequence. Five of these motifs are occupied in ENCODE transcription factor ChIP seq analyses; ESR1.1, ESR1.2, PAX5, REST, and two SP1.2 sites. *PKM* gene expression is activated by SP1 in response to insulin (Yamada and Noguchi, 1999; Yamada et al., 2000). In MCF7 cells *PKM* is highly expressed in the absence of estradiol, and it is regulated by estradiol, expression is reduced by∼50% 40 min after exposure to estradiol (Barrett et al., 2013; Hah et al., 2011) (GEO accession number 27463).

The TOP2B peak on the *RALGAPA2* (Ral GTPase activating protein, alpha subunit 2) promoter also overlaps with a CpG island, ERα and RNA pol II ChIP seq peaks. The TOP2B peak on *RALGAPA2* has 14 of the enriched transcription factor motifs within its sequence, four of these are occupied in encode data ESR1.1 and ESR1.2 and two SP1.2 sites. RALGAPA2 is a substrate for insulin activated protein kinase AKT and has similarities to the tuberous sclerosis complex (Gridley et al., 2005) RALGAPA2 is expressed in MCF7 cells in the absence of estradiol and is regulated by estradiol, expression is increased >3 fold at 40 min (Hah et al., 2011) (GEO accession number 27463).

The TOP2B peak on the *RHOD* promoter also overlaps with a CpG island, ERα and RNA pol II ChIP seq peaks. The sequence bound by TOP2B on the *RHOD* promoter contains fifteen motifs for enriched transcription factors. The CTCF site is occupied in encode data. *RHOD* is one of the genes in the KEGG axon guidance pathway. *RHOD* is expressed in MCF7 cells in the absence of estradiol and is regulated by estradiol, expression increased >4 fold at 40 min (Hah et al., 2011) (GEO accession number 27463).

## DISCUSSION

It is well established that topoisomerases are part of the overall machinery required for efficient transcription, regulating negative superhelical torsion upstream and positive torsion ahead of an elongating polymerase, as outlined in the twin domain model (Liu and Wang, 1987; Brill et al., 1987; Egyházi and Durban, 1987; Schultz et al., 1992; Mondal and Parvin, 2001; Mondal et al., 2003; Salceda et al., 2006; French et al., 2011; Joshi et al., 2012; Kouzine et al., 2013, 2014). Transcription and topoisomerase activity also appear to work in concert to generate large-scale genomic topological domains (Naughton et al., 2013). More recently, evidence has come to light suggesting that TOP2B has a role in regulating transcription of particular sets of genes. Specifically, *Top2b* is required for normal neural development (Yang et al., 2000; Nur-E-Kamal et al., 2007; Lyu et al., 2006; Tiwari et al., 2012) and TOP2B has been demonstrated to be recruited to certain hormone responsive promoters upon ligand treatment (Haffner et al., 2010; Ju et al., 2006; Wong et al., 2009; Perillo et al., 2008). TOP2B negatively regulates the expression of RARα following exposure to retinoic acid (McNamara et al., 2008) and was isolated as a component of the Groucho/TLE repressor complex, along with PARP1 (Ju et al., 2004). The extracellular ligands appear to trigger a molecular switch/change of chromatin state via TOP2B. TOP2B is a component of multi protein complexes that mediates ligand induced transcriptional responses. PARP1 and DNA-PK are often present in these complexes. The involvement of TOP2B in both transcriptional activation and repression is also supported by mouse brain development studies, where both increases and decreases in transcription of developmentally regulated genes where found in the late stages of neuronal differentiation in the absence of Top2b (Lyu et al., 2006; Tiwari et al., 2012). Thus, for certain genes at least, TOP2B appears to be required for the full range of transcriptional response to ligand-induced transcriptional or differentiation-associated stimuli.

To determine whether TOP2 recruitment is a common feature of estradiol-regulated genes we carried out ChIP-SEQ with anti-TOP2B antibodies in human MCF7 cells before and after estradiol treatment (10 nM) for 30 min to visualise the distribution of TOP2B in the human genome (Ju et al., 2006; Perillo et al., 2008). Peaks of binding were identified using three peak calling methods, manual, MACS and SISSRS methods, peak sets identified by at least two methods (TOP2B^2^) were selected for further analysis. The distribution of TOP2B peaks is shown in Fig. 2, TOP2B peaks are distributed throughout the genome and their location is dynamic, as can be seen in Fig. 2C. Two percent of the TOP2B^2^ peaks fall within 5 kb upstream of an annotated TSS and 48% fall within genes is shown in Fig. 2D. Peaks were also analysed for their coincidence with transcriptional markers (Fig. 3; Fig. S2). Two percent of the TOP2B^2^ peak set coincided with H3K4Me3 marks in untreated cells and this reduced >4 fold in estradiol treated cells, consistent with reduced TOP2B occupancy at active promoters and CpG islands following estradiol treatment (Fig. 3; Fig. S2). In this first whole genome TOP2B analysis, we show that peaks of occupancy are distributed across the whole genome, and are not located specifically in promoter regions.

Published GroSEQ data for MCF7 cells treated with 100 nM estradiol for 10, 40 and 160 min was utilised to determine which genes are expressed in these cells and which change in response to estradiol (Hah et al., 2011). Intersects between these gene sets and TOP2B peaks has been used to determine which genes are expressed in MCF7 and which ones are regulated by estradiol in MCF7, this data is shown for the TOP2B^2^ data sets in Table S2. Twenty one percent of the non-expressed genes were associated with TOP2B peaks whilst 32% of expressed genes were associated with TOP2B peaks. So expressed genes were more frequently associated with TOP2B peaks than non-expressed genes (32% and 21% respectively) (Table S3).When expressed genes were divided into those that changed in response to estradiol and those that did not, 30% of the genes that did not change in response to estradiol had TOP2B peaks and 34% of those that changed had TOP2B peaks. Interestingly of the transcripts that change in level following exposure to estradiol more transcripts go down than up, consistent with estradiol being able to both repress and activate gene expression (Table S4).

Gene ontology enrichment analysis highlighted numerous signalling pathways in the molecular functions (Fig. S3) including protein tyrosine kinase activity, ion channel activity, GTPase regulator activity and glutamate receptor activity. Consistent with these molecular functions, cellular response to stimulus, regulation of signal transduction and glutamate receptor signalling were highly enriched in the biological processes. The enriched cellular components were consistent with the previously described role in neuronal development (Yang et al., 2000; Nur-E-Kamal et al., 2007; Tiwari et al., 2012; Capranico et al., 1992; Lyu and Wang, 2003; King et al., 2013; Sano et al., 2008; Nevin et al., 2011). Enriched KEGG pathways included multiple signalling pathways including the ErbB signalling pathway and calcium signalling. Since TOP2B plays a role in neural development we looked for overlaps between our gene lists and those genes implicated in the development of autism and schizophrenia, the overlaps are shown in Fig. 4 and Table S1. One gene that is linked to both autism and schizophrenia with an associated TOP2B peak is the calcium channel protein *CACNA1C* (Bhat et al., 2012; Giusti-Rodríguez and Sullivan, 2013).

Axon guidance was a significantly enriched KEGG pathway. The top2b knockout mouse has an innervation problem, failing to develop the dendrities between the nerve and the muscle cell at the neuromuscular junction, indicative of an axon guidance error. Notably the zebrafish mutant *notorious* (*noto*) lacks functional top2b and displays neurite targeting defects in the retinal ganglion. Specific heparin sulphate structures are involved in axon guidance of retinal ganglion cells (Irie et al., 2002; Merry and Wilson, 2002). Notably, the KEGG pathway for glycosaminoglycan biosynthesis−heparan sulphate/heparin pathway is enriched in our data set which suggests the possibility that topoisomerase regulation of expression of enzymes that transfer sulphates onto heparan could underlie the axon guidance errors seen in the zebrafish mutant *noto* and in the top2b knockout mouse.

## MATERIALS AND METHODS

### Cell culture

MCF7 cells were cultured in Minimum Essential Medium Eagle (MEME) (Sigma-Aldrich, M2279) supplemented with 10% (v/v) foetal bovine serum (PAA, A15-151), 1% (v/v) penicillin and streptomycin (PAA, P11-010) and 1% (v/v) glutamine (200 mM) (GIBCO, 25030-032), 1% NEAA (Sigma-Aldrich, M7145) in a 37°C incubator with 5% CO_2_. Estradiol (E2) was dissolved in the above medium at a stock concentration of 20 µg/ml and for E2 treatment was added to cells to a final concentration of 10 nM for 30 min.

### Antibody purification

Rabbit polyclonal antisera 3535 and 30400 were raised in-house (Cowell et al., 2012) to recombinant human TOP2B C-terminal domain, and the IgG fraction purified on protein A sepharose beads. Western blots with these antibodies are shown in Fig. S1.

### Chromatin immunoprecipitation (ChIP)

ChIP was performed using the kits and the protocol from Millipore (Magna ChIP A, 17-610/EZ-Magna ChIP A, 17-408). Briefly, MCF7 cells were grown to ∼80% confluency and treated with 17β-estradiol (10 nM, 30 min) (Sigma-Aldrich). E2-treated and untreated (control) cells were fixed with formaldehyde (1% (v/v), Sigma-Aldrich), neutralised with glycine, washed with 1× PBS and trypsinised (0.05% trypsin in 0.53 mM EDTA (Invitrogen, 25300-054). Cells were collected and lysed with cell lysis buffer containing protease inhibitors, collected and resuspended in nuclear lysis buffer containing protease inhibitors. Chromatin was sonicated to between 500-1000 bp, 14×15 s cycles, and 5% was removed as ‘input’. 3.6 µg anti-TOP2B (3535), 7 µg anti-TOP2B (30400), or 1 µg anti-GFP (Santa Cruz Biotechnology, sc-8334) or 5 µg anti-AcH3 (Millipore, 06-599B) were added to protein A magnetic beads (Invitrogen, 100.02D). Chromatin was diluted, incubated with the beads and rotated for 4.5 h at 4°C. Immunoprecipitated protein-DNA complexes were washed sequentially in Low Salt Buffer, High Salt Buffer, LiCl Buffer and TE Buffer and then resuspended in ChIP Elution Buffer+Proteinase K, rotated at 65°C for 2 h followed by incubation at 95°C for 10 min. DNA was then purified using spin columns (Binding Reagent ‘A’, Wash Buffer ‘B’, Elution reagent ‘C’).

### Quantitative real time PCR (qPCR)

qPCR was performed using iQ SYBR green supermix (Bio-Rad, 10003253), primers including GAPDH control primers are shown in the Table S1. qPCR parameters were −95°C for 5 min (1 cycle), 95°C for 25 s, then 60°C for 25 s, then 72°C for 10 s (40 cycles), 72°C for 6 min (1 cycle). Results were quantified as % input with GFP and AcH3% input values being the negative and positive controls respectively.

### ChIP Seq

Libraries were generated with chromatin immunoprecipitated from MCF7 cells±E2 (10 nM for 30 min). Multiple immunoprecipitates were pooled for each antibody (3535, 30400 and anti-GFP) to obtain between 400 and 800 ng DNA, at a concentration of 6-21 ng/µl for library preparation for Illumina ChIP Sequencing on an Illumina Genome Analyser II. Each library was sequenced 3 or 4 times for input libraries and for TOP2B libraries. The data from the 3535 and 30400 libraries was merged to give >10 million uniquely aligned reads. Genome Alignment, the raw sequencing reads were aligned to the human reference genome (hg18) using BWA (v0.5.8c) (Li and Durbin, 2009) and alignment files were converted to BED format using BEDTools (v2.12.0) (Quinlan and Hall, 2010). BED files were used for peak calling by MACS (v1.4) (Zhang et al., 2008) and SISSRs (Jothi et al., 2008) using INPUT controls as background. Manual peak calling was carried out by visual inspection of aligned sequence reads using Genome Studio software (2008.1 Framework), employing input and GFP lanes as controls. Overlapping peak sets were generated using bedtools intersect, and features coincident with consensus peaks (gene neighbourhood, TSS, transcriptional markers), were also found using bedtools intersect. Data is deposited in GEO, accession number GSE66753.

### Gene ontology and KEGG pathway enrichment

Gene ontology and KEGG pathway enrichment analysis was carried out using GOstats (v 2.24.0) (Falcon and Gentleman, 2007) Bioconductor package. Enrichment was then calculated by applying a hypergeometric test and correcting for multiple tests as per Benjamini-Hochberg (Benjamini and Hochberg, 1995). GO terms are reported as significant if they have a corrected *P*<0.01.

### Transcription factor motifs

Vertebrate transcription factor motifs were obtained in MEME format from the Jaspar database (2014 release) (Mathelier et al., 2013). FIMO (v 4.9.1), from the MEME suite (Bailey et al., 2009; Grant et al., 2011), was used to find occurrences of motifs in the consensus peak sets and in the whole reference genome. Binding site enrichment was then calculated by applying a hypergeometric test and correcting for multiple tests as per Benjamini-Hochberg (Benjamini and Hochberg, 1995). Motifs are reported as significant if they have a corrected *P*<0.05. A distance measure for binary attributes and Ward's method was used for clustering of genes and transcription factor motifs associated with TOP2B peaks. All heatmaps were generated with the gplots v 2.12.1 BioConductor package.

## References

[BIO014308C1] Akimitsu N., Kamura K., Toné S., Sakaguchi A., Kikuchi A., Hamamoto H. and Sekimizu K. (2003). Induction of apoptosis by depletion of DNA topoisomerase IIα in mammalian cells. *Biochem. Biophys. Res. Commun.* 307, 301-307. 10.1016/S0006-291X(03)01169-0 12859955

[BIO014308C2] Amsterdam A., Nissen R. M., Sun Z., Swindell E. C., Farrington S. and Hopkins N. (2004). Identification of 315 genes essential for early zebrafish development. *Proc. Natl. Acad. Sci. USA* 101, 12792-12797. 10.1073/pnas.0403929101 15256591 PMC516474

[BIO014308C3] Antequera F. and Bird A. (1993). Number of CpG islands and genes in human and mouse. *Proc. Natl. Acad. Sci. USA* 90, 11995-11999. 10.1073/pnas.90.24.11995 7505451 PMC48112

[BIO014308C4] Austin C. A. and Marsh K. L. (1998). Eukaryotic DNA topoisomerase IIβ. *BioEssays* 20, 215-226. 10.1002/(SICI)1521-1878(199803)20:3<215::AID-BIES5>3.0.CO;2-Q 9631649

[BIO014308C84] Bailey T. L., Boden M., Buske F. A., Frith M., Grant C. E., Clementi L., Ren J., Li W. W. and Noble W. S. (2009). MEME Suite: tools for motif discovery and searching. *Nucleic Acids Res.* 37, W202-W208. 10.1093/nar/gkp335 19458158 PMC2703892

[BIO014308C85] Barrett T., Wilhite S. E., Ledoux P., Evangelista C., Kim I. F., Tomashevsky M., Marshall K. A., Phillippy K. H., Sherman P. M., Holko M., Yefanov A., Lee H., Zhang N., Robertson C. L., Serova N., Davis S. and Soboleva A. (2013). NCBI GEO: archive for functional genomics data sets–update. *Nucleic Acids Res.* 41, D991-D995. 10.1093/nar/gks1193 23193258 PMC3531084

[BIO014308C5] Basu S. N., Kollu R. and Banerjee-Basu S. (2009). AutDB: a gene reference resource for autism research. *Nucleic Acids Res.* 37, D832-D836. 10.1093/nar/gkn835 19015121 PMC2686502

[BIO014308C6] Benjamini Y. and Hochberg Y. (1995). A practical and powerful approach to multiple testing. *J. R. Stat. Soc. Ser. B Methodol.* 57, 289-300.

[BIO014308C7] Bertelsen B., Melchior L., Jensen L. R., Groth C., Glenthøj B., Rizzo R., Debes N. M., Skov L., Brøndum-Nielsen K., Paschou P. et al. (2014). Intragenic deletions affecting two alternative transcripts of the IMMP2L gene in patients with Tourette syndrome. *Eur. J. Hum. Genet.* 22, 1283-1289. 10.1038/ejhg.2014.24 24549057 PMC4200436

[BIO014308C8] Bhat S., Dao D. T., Terrillion C. E., Arad M., Smith R. J., Soldatov N. M. and Gould T. D. (2012). CACNA1C (Cav1.2) in the pathophysiology of psychiatric disease. *Prog. Neurobiol.* 99, 1-14. 10.1016/j.pneurobio.2012.06.001 22705413 PMC3459072

[BIO014308C9] Brill S. J., DiNardo S., Voelkel-Meiman K. and Sternglanz R. (1987). Need for DNA topoisomerase activity as a swivel for DNA replication for transcription of ribosomal RNA. *Nature* 326, 414-416. 10.1038/326414a0 2436053

[BIO014308C10] Capranico G., Tinelli S., Austin C. A., Fisher M. L. and Zunino F. (1992). Different patterns of gene expression of topoisomerase II isoforms in differentiated tissues during murine development. *Biochim. Biophys. Acta* 1132, 43-48. 10.1016/0167-4781(92)90050-A 1380833

[BIO014308C11] Carroll J. S., Meyer C. A., Song J., Li W., Geistlinger T. R., Eeckhoute J., Brodsky A. S., Keeton E. K., Fertuck K. C., Hall G. F. et al. (2006). Genome-wide analysis of estrogen receptor binding sites. *Nat. Genet.* 38, 1289-1297. 10.1038/ng1901 17013392

[BIO014308C12] Cowell I. G., Okorokov A. L., Cutts S. A., Padget K., Bell M., Milner J. and Austin C. A. (2000). Human topoisomerase IIα and IIβ interact with the C-terminal region of p53. *Exp. Cell Res.* 255, 86-94. 10.1006/excr.1999.4772 10666337

[BIO014308C13] Cowell I. G., Papageorgiou N., Padget K., Watters G. P. and Austin C. A. (2011). Histone deacetylase inhibition redistributes topoisomerase IIβ from heterochromatin to euchromatin. *Nucleus* 2, 61-71. 10.4161/nucl.14194 21647300 PMC3104810

[BIO014308C14] Cowell I. G., Sondka Z., Smith K., Lee K. C., Manville C. M., Sidorczuk-Lesthuruge M., Rance H. A., Padget K., Jackson G. H., Adachi N. et al. (2012). Model for MLL translocations in therapy-related leukemia involving topoisomerase IIβ-mediated DNA strand breaks and gene proximity. *Proc. Natl. Acad. Sci. USA* 109, 8989-8994. 10.1073/pnas.1204406109 22615413 PMC3384169

[BIO014308C15] Dastidar S. G., Narayanan S., Stifani S. and D'Mello S. R. (2012). Transducin-like enhancer of Split-1 (TLE1) combines with Forkhead box protein G1 (FoxG1) to promote neuronal survival. *J. Biol. Chem.* 287, 14749-14759. 10.1074/jbc.M111.328336 22354967 PMC3340250

[BIO014308C16] De Rubeis S., He X., Goldberg A. P., Poultney C. S., Samocha K., Ercument Cicek A., Kou Y., Liu L., Fromer M., Walker S. et al. (2014). Synaptic, transcriptional and chromatin genes disrupted in autism. *Nature* 515, 209-215. 10.1038/nature13772 25363760 PMC4402723

[BIO014308C17] Dereuddre S., Delaporte C. and Jacquemin-Sablon A. (1997). Role of topoisomerase IIβ in the resistance of 9-OH-ellipticine-resistant Chinese hamster fibroblasts to topoisomerase II inhibitors. *Cancer Res.* 57, 4301-4308. 9331091

[BIO014308C18] Dovey M., Patton E. E., Bowman T., North T., Goessling W., Zhou Y. and Zon L. I. (2009). Topoisomerase IIα is required for embryonic development and liver regeneration in zebrafish. *Mol. Cell. Biol.* 29, 3746-3753. 10.1128/MCB.01684-08 19380487 PMC2698760

[BIO014308C19] Egyházi E. and Durban E. (1987). Microinjection of anti-topoisomerase I immunoglobulin G into nuclei of Chironomus tentans salivary gland cells leads to blockage of transcription elongation. *Mol. Cell. Biol.* 7, 4308-4316. 2449604 10.1128/mcb.7.12.4308-4316.1987 PMC368113

[BIO014308C20] Falcon S. and Gentleman R. (2007). Using GOstats to test gene lists for GO term association. *Bioinformatics* 23, 257-258. 10.1093/bioinformatics/btl567 17098774

[BIO014308C21] French S. L., Sikes M. L., Hontz R. D., Osheim Y. N., Lambert T. E., El Hage A., Smith M. M., Tollervey D., Smith J. S. and Beyer A. L. (2011). Distinguishing the roles of Topoisomerases I and II in relief of transcription-induced torsional stress in yeast rRNA genes. *Mol. Cell. Biol.* 31, 482-494. 10.1128/MCB.00589-10 21098118 PMC3028620

[BIO014308C22] Gimelli S., Capra V., Di Rocco M., Leoni M., Mirabelli-Badenier M., Schiaffino M. C., Fiorio P., Cuoco C., Gimelli G. and Tassano E. (2014). Interstitial 7q31.1 copy number variations disrupting IMMP2L gene are associated with a wide spectrum of neurodevelopmental disorders. *Mol. Cytogenet.* 7, 54 10.1186/s13039-014-0054-y 25478008 PMC4255718

[BIO014308C23] Giusti-Rodríguez P. and Sullivan P. F. (2013). The genomics of schizophrenia: update and implications. *J. Clin. Invest.* 123, 4557-4563. 10.1172/JCI66031 24177465 PMC3809776

[BIO014308C24] Grant C. E., Bailey T. L. and Noble W. S. (2011). FIMO: scanning for occurrences of a given motif. *Bioinformatics* 27, 1017-1018. 10.1093/bioinformatics/btr064 21330290 PMC3065696

[BIO014308C25] Gridley S., Lane W. S., Garner C. W. and Lienhard G. E. (2005). Novel insulin-elicited phosphoproteins in adipocytes. *Cell. Signal.* 17, 59-66. 10.1016/j.cellsig.2004.05.013 15451025

[BIO014308C26] Haffner M. C., Aryee M. J., Toubaji A., Esopi D. M., Albadine R., Gurel B., Isaacs W. B., Bova G. S., Liu W., Xu J. et al. (2010). Androgen-induced TOP2B-mediated double-strand breaks and prostate cancer gene rearrangements. *Nat. Genet.* 42, 668-675. 10.1038/ng.613 20601956 PMC3157086

[BIO014308C27] Hah N., Danko C. G., Core L., Waterfall J. J., Siepel A., Lis J. T. and Kraus W. L. (2011). A rapid, extensive, and transient transcriptional response to estrogen signaling in breast cancer cells. *Cell* 145, 622-634. 10.1016/j.cell.2011.03.042 21549415 PMC3099127

[BIO014308C28] Hedges V. L., Ebner T. J., Meisel R. L. and Mermelstein P. G. (2012). The cerebellum as a target for estrogen action. *Front. Neuroendocrinol.* 33, 403-411. 10.1016/j.yfrne.2012.08.005 22975197 PMC3496070

[BIO014308C29] Heng X., Jin G., Zhang X., Yang D., Zhu M., Fu S., Li X. and Le W. (2012). Nurr1 regulates Top IIβ and functions in axon genesis of mesencephalic dopaminergic neurons. *Mol. Neurodegener.* 7, 4 10.1186/1750-1326-7-4 22296971 PMC3359158

[BIO014308C30] Irie A., Yates E. A., Turnbull J. E. and Holt C. E. (2002). Specific heparan sulfate structures involved in retinal axon targeting. *Development* 129, 61-70. 11782401 10.1242/dev.129.1.61

[BIO014308C31] Johnson C. A., Padget K., Austin C. A. and Turner B. M. (2001). Deacetylase activity associates with topoisomerase II and is necessary for etoposide-induced apoptosis. *J. Biol. Chem.* 276, 4539-4542. 10.1074/jbc.C000824200 11136718

[BIO014308C32] Joseph R., Orlov Y. L., Huss M., Sun W., Kong S. L., Ukil L., Pan Y. F., Li G., Lim M., Thomsen J. S. et al. (2010). Integrative model of genomic factors for determining binding site selection by estrogen receptor-α. *Mol. Syst. Biol.* 6, 456 10.1038/msb.2010.109 21179027 PMC3018168

[BIO014308C33] Joshi R. S., Piña B. and Roca J. (2012). Topoisomerase II is required for the production of long Pol II gene transcripts in yeast. *Nucleic Acids Res.* 40, 7907-7915. 10.1093/nar/gks626 22718977 PMC3439932

[BIO014308C34] Jothi R., Cuddapah S., Barski A., Cui K. and Zhao K. (2008). Genome-wide identification of in vivo protein-DNA binding sites from ChIP-Seq data. *Nucleic Acids Res.* 36, 5221-5231. 10.1093/nar/gkn488 18684996 PMC2532738

[BIO014308C35] Ju B.-G., Solum D., Song E. J., Lee K.-J., Rose D. W., Glass C. K. and Rosenfeld M. G. (2004). Activating the PARP-1 sensor component of the Groucho/TLE1 corepressor complex mediates a CaMKinase IIδ-dependent neurogenic gene activation pathway. *Cell* 119, 815-829. 10.1016/j.cell.2004.11.017 15607978

[BIO014308C36] Ju B.-G., Lunyak V. V., Perissi V., Garcia-Bassets I., Rose D. W., Glass C. K. and Rosenfeld M. G. (2006). A topoisomerase IIβ-mediated dsDNA break required for regulated transcription. *Science* 312, 1798-1802. 10.1126/science.1127196 16794079

[BIO014308C37] King I. F., Yandava C. N., Mabb A. M., Hsiao J. S., Huang H.-S., Pearson B. L., Calabrese J. M., Starmer J., Parker J. S., Magnuson T. et al. (2013). Topoisomerases facilitate transcription of long genes linked to autism. *Nature* 501, 58-62. 10.1038/nature12504 23995680 PMC3767287

[BIO014308C38] Kouzine F., Gupta A., Baranello L., Wojtowicz D., Ben-Aissa K., Liu J., Przytycka T. M. and Levens D. (2013). Transcription-dependent dynamic supercoiling is a short-range genomic force. *Nat. Struct. Mol. Biol.* 20, 396-403. 10.1038/nsmb.2517 23416947 PMC3594045

[BIO014308C39] Kouzine F., Levens D. and Baranello L. (2014). DNA topology and transcription. *Nucleus* 5, 195-202. 10.4161/nucl.28909 24755522 PMC4133214

[BIO014308C40] Lee M. P., Sander M. and Hsieh T. (1989). Nuclease protection by Drosophila DNA topoisomerase II. Enzyme/DNA contacts at the strong topoisomerase II cleavage sites. *J. Biol. Chem.* 264, 21779-21787. 2557338

[BIO014308C41] LeRoy G., Loyola A., Lane W. S. and Reinberg D. (2000). Purification and characterization of a human factor that assembles and remodels chromatin. *J. Biol. Chem.* 275, 14787-14790. 10.1074/jbc.C000093200 10747848

[BIO014308C42] Letovsky J. and Dynan W. S. (1989). Measurement of the binding of transcription factor Sp1 to a single GC box recognition sequence. *Nucleic Acids Res.* 17, 2639-2653. 10.1093/nar/17.7.2639 2717405 PMC317648

[BIO014308C43] Li H. and Durbin R. (2009). Fast and accurate short read alignment with Burrows–Wheeler transform. *Bioinformatics* 25, 1754-1760. 10.1093/bioinformatics/btp324 19451168 PMC2705234

[BIO014308C44] Lin C.-Y., Vega V. B., Thomsen J. S., Zhang T., Kong S. L., Xie M., Chiu K. P., Lipovich L., Barnett D. H., Stossi F. et al. (2007). Whole-genome cartography of estrogen receptor alpha binding sites. *PLoS Genet.* 3, e87 10.1371/journal.pgen.0030087 17542648 PMC1885282

[BIO014308C45] Liu L. F. and Wang J. C. (1987). Supercoiling of the DNA template during transcription. *Proc. Natl. Acad. Sci. USA* 84, 7024-7027. 10.1073/pnas.84.20.7024 2823250 PMC299221

[BIO014308C46] Lyu Y. L. and Wang J. C. (2003). Aberrant lamination in the cerebral cortex of mouse embryos lacking DNA topoisomerase IIβ. *Proc. Natl. Acad. Sci. USA* 100, 7123-7128. 10.1073/pnas.1232376100 12773624 PMC165840

[BIO014308C47] Lyu Y. L., Lin C.-P., Azarova A. M., Cai L., Wang J. C. and Liu L. F. (2006). Role of topoisomerase IIβ in the expression of developmentally regulated genes. *Mol. Cell. Biol.* 26, 7929-7941. 10.1128/MCB.00617-06 16923961 PMC1636731

[BIO014308C48] Mao Y., Desai S. D. and Liu L. F. (2000). SUMO-1 conjugation to human DNA topoisomerase II isozymes. *J. Biol. Chem.* 275, 26066-26073. 10.1074/jbc.M001831200 10862613

[BIO014308C49] Mathelier A., Zhao X., Zhang A. W., Parcy F., Worsley-Hunt R., Arenillas D. J., Buchman S., Chen C.-Y., Chou A., Ienasescu H. et al. (2013). JASPAR 2014: an extensively expanded and updated open-access database of transcription factor binding profiles. *Nucleic Acids Res.* 42, D142-D147. 10.1093/nar/gkt997 24194598 PMC3965086

[BIO014308C50] McNamara S., Wang H., Hanna N. and Miller W. H. (2008). Topoisomerase IIβ negatively modulates retinoic acid receptor α function: a novel mechanism of retinoic acid resistance. *Mol. Cell. Biol.* 28, 2066-2077. 10.1128/MCB.01576-07 18212063 PMC2268389

[BIO014308C51] Meitzen J. and Mermelstein P. G. (2011). Estrogen receptors stimulate brain region specific metabotropic glutamate receptors to rapidly initiate signal transduction pathways. *J. Chem. Neuroanat.* 42, 236-241. 10.1016/j.jchemneu.2011.02.002 21458561 PMC3146967

[BIO014308C52] Merry C. L. R. and Wilson V. A. (2002). Role of heparan sulfate-2-O-sulfotransferase in the mouse. *Biochim. Biophys. Acta* 1573, 319-327. 10.1016/S0304-4165(02)00399-9 12417414

[BIO014308C53] Mondal N. and Parvin J. D. (2001). DNA topoisomerase IIα is required for RNA polymerase II transcription on chromatin templates. *Nature* 413, 435-438. 10.1038/35096590 11574892

[BIO014308C54] Mondal N., Zhang Y., Jonsson Z., Dhar S. K., Kannapiran M. and Parvin J. D. (2003). Elongation by RNA polymerase II on chromatin templates requires topoisomerase activity. *Nucleic Acids Res.* 31, 5016-5024. 10.1093/nar/gkg705 12930951 PMC212805

[BIO014308C55] Nakano H., Yamazaki T., Miyatake S., Nozaki N., Kikuchi A. and Saito T. (1996). Specific interaction of topoisomerase IIβ and the CD3ε chain of the T cell receptor complex. *J. Biol. Chem.* 271, 6483-6489. 10.1074/jbc.271.11.6483 8626450

[BIO014308C56] Naughton C., Avlonitis N., Corless S., Prendergast J. G., Mati I. K., Eijk P. P., Cockroft S. L., Bradley M., Ylstra B. and Gilbert N. (2013). Transcription forms and remodels supercoiling domains unfolding large-scale chromatin structures. *Nat. Struct. Mol. Biol.* 20, 387-395. 10.1038/nsmb.2509 23416946 PMC3689368

[BIO014308C57] Nevin L. M., Xiao T., Staub W. and Baier H. (2011). Topoisomerase IIβ is required for lamina-specific targeting of retinal ganglion cell axons and dendrites. *Development* 138, 2457-2465. 10.1242/dev.060335 21610027 PMC3100707

[BIO014308C58] Nur-E-Kamal A., Meiners S., Ahmed I., Azarova A., Lin C.-P., Lyu Y. L. and Liu L. F. (2007). Role of DNA topoisomerase IIβ in neurite outgrowth. *Brain Res.* 1154, 50-60. 10.1016/j.brainres.2007.04.029 17493591

[BIO014308C59] Papantonis A. and Cook P. R. (2013). Transcription factories: genome organization and gene regulation. *Chem. Rev.* 113, 8683-8705. 10.1021/cr300513p. 23597155

[BIO014308C60] Perillo B., Ombra M. N., Bertoni A., Cuozzo C., Sacchetti S., Sasso A., Chiariotti L., Malorni A., Abbondanza C. and Avvedimento E. V. (2008). DNA oxidation as triggered by H3K9me2 demethylation drives estrogen-induced gene expression. *Science* 319, 202-206. 10.1126/science.1147674 18187655

[BIO014308C61] Quinlan A. R. and Hall I. M. (2010). BEDTools: a flexible suite of utilities for comparing genomic features. *Bioinformatics* 26, 841-842. 10.1093/bioinformatics/btq033 20110278 PMC2832824

[BIO014308C62] Salceda J., Fernández X. and Roca J. (2006). Topoisomerase II, not topoisomerase I, is the proficient relaxase of nucleosomal DNA. *EMBO J.* 25, 2575-2583. 10.1038/sj.emboj.7601142 16710299 PMC1478187

[BIO014308C63] Sano K., Miyaji-Yamaguchi M., Tsutsui K. M. and Tsutsui K. (2008). Topoisomerase IIβ activates a subset of neuronal genes that are repressed in AT-rich genomic environment. *PLoS ONE* 3, e4103 10.1371/journal.pone.0004103 19116664 PMC2605559

[BIO014308C64] Sapetto-Rebow B., McLoughlin S. C., O'Shea L. C., O'Leary O., Willer J. R., Alvarez Y., Collery R., O'Sullivan J., Van Eeden F., Hensey C. et al. (2011). Maternal topoisomerase IIα, not topoisomerase IIβ, enables embryonic development of zebrafish top2a−/− mutants. *BMC Dev. Biol.* 11, 71 10.1186/1471-213X-11-71 22111588 PMC3287258

[BIO014308C65] Schizophrenia Working Group of the Psychiatric Genomics Consortium (2014). Biological insights from 108 schizophrenia-associated genetic loci. *Nature* 511, 421-427. 10.1038/nature13595 25056061 PMC4112379

[BIO014308C66] Schultz M. C., Brill S. J., Ju Q., Sternglanz R. and Reeder R. H. (1992). Topoisomerases and yeast rRNA transcription: negative supercoiling stimulates initiation and topoisomerase activity is required for elongation. *Genes Dev.* 6, 1332-1341. 10.1101/gad.6.7.1332 1321070

[BIO014308C67] Thomsen B., Bendixen C., Lund K., Andersen A. H., Sørensen B. S. and Westergaard O. (1990). Characterization of the interaction between topoisomerase II and DNA by transcriptional footprinting. *J. Mol. Biol.* 215, 237-244. 10.1016/S0022-2836(05)80342-0 2170662

[BIO014308C68] Tiwari V. K., Burger L., Nikoletopoulou V., Deogracias R., Thakurela S., Wirbelauer C., Kaut J., Terranova R., Hoerner L., Mielke C. et al. (2012). Target genes of Topoisomerase IIβ regulate neuronal survival and are defined by their chromatin state. *Proc. Natl. Acad. Sci. USA* 109, E934-E943. 10.1073/pnas.1119798109 22474351 PMC3340998

[BIO014308C69] Tsai S. C., Valkov N., Yang W. M., Gump J., Sullivan D. and Seto E. (2000). Histone deacetylase interacts directly with DNA topoisomerase II. *Nat. Genet.* 26, 349-353. 10.1038/81671 11062478

[BIO014308C70] Tsutsui K., Tsutsui K., Okada S., Watanabe M., Shohmori T., Seki S. and Inoue Y. (1993). Molecular cloning of partial cDNAs for rat DNA topoisomerase II isoforms and their differential expression in brain development. *J. Biol. Chem.* 268, 19076-19083. 8395528

[BIO014308C71] Tsutsui K., Tsutsui K., Sano K., Kikuchi A. and Tokunaga A. (2001). Involvement of DNA topoisomerase IIβ in neuronal differentiation. *J. Biol. Chem.* 276, 5769-5778. 10.1074/jbc.M008517200 11106659

[BIO014308C72] Wendorff T. J., Schmidt B. H., Heslop P., Austin C. A. and Berger J. M. (2012). The structure of DNA-bound human topoisomerase II alpha: conformational mechanisms for coordinating inter-subunit interactions with DNA cleavage. *J. Mol. Biol.* 424, 109-124. 10.1016/j.jmb.2012.07.014 22841979 PMC3584591

[BIO014308C73] West K. L. and Austin C. A. (1999). Human DNA topoisomerase IIβ binds and cleaves four-way junction DNA in vitro. *Nucleic Acids Res.* 27, 984-992. 10.1093/nar/27.4.984 9927730 PMC148277

[BIO014308C74] Wong R. H. F., Chang I., Hudak C. S. S., Hyun S., Kwan H.-Y. and Sul H. S. (2009). A role of DNA-PK for the metabolic gene regulation in response to insulin. *Cell* 136, 1056-1072. 10.1016/j.cell.2008.12.040 19303849 PMC2768498

[BIO014308C75] Wu C.-C., Li T.-K., Farh L., Lin L.-Y., Lin T.-S., Yu Y.-J., Yen T.-J., Chiang C.-W. and Chan N.-L. (2011). Structural basis of type II topoisomerase inhibition by the anticancer drug etoposide. *Science* 333, 459-462. 10.1126/science.1204117 21778401

[BIO014308C76] Xiao H. and Goodrich D. W. (2005). The retinoblastoma tumor suppressor protein is required for efficient processing and repair of trapped topoisomerase II-DNA-cleavable complexes. *Oncogene* 24, 8105-8113. 10.1038/sj.onc.1208958 16091739 PMC2799250

[BIO014308C77] Yamada K. and Noguchi T. (1999). Nutrient and hormonal regulation of pyruvate kinase gene expression. *Biochem. J.* 337, 1-11. 10.1042/bj3370001 9854017 PMC1219928

[BIO014308C78] Yamada K., Tanaka T., Miyamoto K. and Noguchi T. (2000). Sp family members and nuclear factor-Y cooperatively stimulate transcription from the rat pyruvate kinase M gene distal promoter region via their direct interactions. *J. Biol. Chem.* 275, 18129-18137. 10.1074/jbc.M001543200 10748033

[BIO014308C79] Yamane K., Kawabata M. and Tsuruo T. (1997). A DNA-topoisomerase-II-binding protein with eight repeating regions similar to DNA-repair enzymes and to a cell-cycle regulator. *Eur. J. Biochem. FEBS* 250, 794-799. 10.1111/j.1432-1033.1997.00794.x 9461304

[BIO014308C80] Yang X., Li W., Prescott E. D., Burden S. J. and Wang J. C. (2000). DNA topoisomerase IIβ and neural development. *Science* 287, 131-134. 10.1126/science.287.5450.131 10615047

[BIO014308C81] Yuwen H., Hsia C. C., Nakashima Y., Evangelista A. and Tabor E. (1997). Binding of wild-type p53 by topoisomerase II and overexpression of topoisomerase II in human hepatocellular carcinoma. *Biochem. Biophys. Res. Commun.* 234, 194-197. 10.1006/bbrc.1997.6539 9168988

[BIO014308C82] Zandvliet D. W. J., Hanby A. M., Austin C. A., Marsh K. L., Clark I. B. N., Wright N. A. and Poulsom R. (1996). Analysis of foetal expression sites of human type II DNA topoisomerase α and β mRNAs by in situ hybridisation. *Biochim. Biophys. Acta* 1307, 239-247. 10.1016/0167-4781(96)00063-2 8679710

[BIO014308C83] Zhang Y., Liu T., Meyer C. A., Eeckhoute J., Johnson D. S., Bernstein B. E., Nusbaum C., Myers R. M., Brown M., Li W. et al. (2008). Model-based analysis of ChIP-Seq (MACS). *Genome Biol.* 9, R137 10.1186/gb-2008-9-9-r137 18798982 PMC2592715

